# Reviews of Books

**Published:** 1924-07

**Authors:** 


### REVIEWS OF BOOKS.

### IMPERIAL HEALTH.

**Health Problems of the Empire.** By Dr. Andrew Balfour, Director of the London School of Hygiene and Tropical Medicine, and Dr. H. H. Scott. (Collins. 16s. net.)

A stage has been reached in the history of the British Commonwealth where the principles of hygiene have received, from those who really count, the adherence to which they are entitled. This is the cheering conclusion reached by the authors of this volume—one of a series of twelve intended to provide a comprehensive survey of the British Empire, its history, development and activities—after they have passed in review much that is dark and disturbing and much that is hopeful and encouraging. They add that no such "crusade" under the banner of health has ever been witnessed as that which is being prosecuted at the present time in wellnigh every portion of the Empire. Laymen and laywomen are encouraged to be of good cheer, and especially those of the devoted band who among the native populations in distant lands are doing their utmost to dispel the clouds of ignorance and superstition. They are reminded that Louis Pasteur was not a physician and that Edwin Chadwick was not qualified in medicine. The lay worker, however, is not alone to be encouraged; the whole community must have its interest aroused and sustained in health matters, not only of the homeland but of the Empire. In this respect, history, simply and graphically presented, plays a great part, and it is the function of the health teacher and lecturer to see that, as thus presented in this book, history and its lessons oust the patent-medicine advertisement from the homes of the people.

The pioneers in medicine and those research workers who have followed in their footsteps have usually required the aid of rude shocks to the community before they could see their labours bearing fruit. Cholera, war, a picturesque death-roll, a spasm of fear—these have proved excellent stimuli to the body politic from time to time. While, for example, in the middle of the nineteenth century, the teaching of Chadwick was beginning to bear fruit, thanks to the stimulus of cholera, what was happening in the Army?—

> Once again it was war which quickened the dry bones, and once again the long and deadening years of peace sapped the soundness of the fabric which had been reared, and in some measure brought about the shame and miseries of the Crimea. Here a mortality of 230 per 1,000 of the strength showed how very little progress, so far as field conditions were concerned, had really been made in Army hygiene, and dysentery and cholera claimed their victims wholesale.

If we come down to the South African War of 1899–1902, we are only at the beginning of the study of disease as regards its actual incidence in the field. The number of deaths (8,000) from typhoid exceeded those (7,000) killed in action:—

> No one who has seen the long line of graves in Bloemfontein cemetery can fail to be impressed and saddened by the ravages of disease in war. Typhoid played the major part, but dysentery, that scourge of armies, also accounted for much invaliding and many deaths, and in those days . . . . there was no means of preventing this disorder.

When the Great War came upon us the Army Medical Service and the medical profession generally faced the position with courage and equanimity and rose to the occasion magnificently. There were some dreadful setbacks in Macedonia, in East Africa, and at first in Mesopotamia. But wonderful victories were won against disease, especially in France and Flanders. Anti-typhoid inoculation was carried out on a huge scale, vaccination against smallpox made compulsory, tetanus wellnigh eliminated by the use of anti-tetanic serum, the virulence of enteric fevers and cholera, where these were not kept entirely in check, notably reduced. Water supplies were purified by chlorination and other means, vermin and the disease-carrying fly combated, foodstuffs protected, billets subjected to sanitary discipline, while mobile hygiene laboratories rushed hither and thither helping the sanitarian to scotch disease at the outset. Nothing like it had ever been witnessed.

As to health conditions at sea, we can but direct attention to what is said in the book itself about the vile conditions of the slave trade or the amazing indecencies of life at sea two hundred years ago. Of the Mercantile Marine the authors say that its record has been deplorable, and that even at the present time much remains to be done. They urge the transfer of the control of the health of this service from the Board of Trade to the Ministry of Health. In the year 1877 Manson, with his scientific demonstration of the activities of the mosquito in the field of disease, laid the foundation for the medical conquest of the tropics. Two years later the London School of Tropical Medicine came into being, although anticipated by a few months by the kindred school of Liverpool, which was wholly due to private enterprise:—

> With Manson as the moving spirit in London and Boyce at the helm in Liverpool, the success of the embryo schools was assured, and they embarked in friendly rivalry on their remarkable careers at the close of a century which had witnessed a vast expansion and a great consolidation of the British Empire.

In a series of chapters devoted, one by one, to our several possessions overseas, the health problems of each colony are examined in this comprehensive book, and the way in which they are being hindered or surmounted set out. Of the West Indies we read of the results of concerted action. In Canada there is no playing second fiddle to any country in public health affairs. There has been a real revolution in sanitation in West Africa in the last five-and-twenty years. In the Union of South Africa there is very much that is disquieting; indeed, a very gloomy picture is drawn of the insanitary conditions, of the carelessness and ignorance of the population, and of death-rates and infant mortality rates far higher than they ought to be. There is now evidence of a more enlightened public opinion; a terrible epidemic of influenza in 1918–19 seems to have acted as a stimulant. The sketch of the fair land of Uganda supplies not only an account of the classic outbreak of human sleeping sickness but some grave figures in one or two other directions. What is to be thought of the following?—

> Venereal disease threatened ruin to the native population. . . . . A change took place in native customs as the result of missionary enterprise and the introduction of European ideas and government. The women, hitherto jealously segregated, were emancipated. . . . A truly appalling state of affairs resulted. . . . The district of Bunyoro in ten years has witnessed a reduction of population from 120,000 to 80,000 due in the main to syphilis.

The urgency of establishing maternity training centres and the need for instruction in child welfare and mother craft are apparent when it is stated that the infantile mortality rates are the highest in the world. It is indeed staggering to read that they vary from 600 to 900 per 1,000. Great Britain does indeed shoulder heavy responsibilities when she extends her territories in Africa. She stands *in loco parentis* to the black races, and the first duty of a parent is to safeguard the health of those who look to her for protection. There is clearly work of a formidable character to be tackled in Uganda. In regard to India, too, we are warned of the risk which medical research runs of being wrecked by the adoption of the recommendations of the Inchcape Commission. The saving of a few lakhs of rupees seems a small matter in a country where it is estimated that six million lives were sacrificed to the plague in 1901–10, four millions from cholera in 1902–08, and six millions from influenza in 1918. As to the great Commonwealth of Australia, one quotation must suffice :—

The mistakes of the past are in process of rectification. A new era has dawned and disease, at least communicable disease, is being mastered and a health record established which may yet excite the envy and admiration of European communities.

These notes must serve as pointers to a volume of absorbing interest.

### THE HEALING SUN.

**Sunlight and Health.** By C. W. Saleeby, M.D., Ch.B., F.Z.S., F.R.S.E. (Nisbet. 5s. net.)

At heart Dr. Saleeby is a journalist of scientific tastes. As a ready and popular writer he is able to impart a vast amount of valuable scientific knowledge to people who would recoil from formal treatises, and as he knows all languages (we suspect him of being fluent in the tongue of the parts of Libya about Cyrene) he can take a wide survey of any subject in which he is interested and tell all about it smoothly and pleasantly. He has long been an exponent of the great things that sunlight can do, and is doing, for the health of the world, and in this readable little volume he is, so to speak, upon his native heath. To some extent it is a volume of reprinted pieces, not too closely co-ordinated, and marked by occasional repetitions ; it is, in fact, a hasty book, but not the less effective for that. Indeed, as a popular account of the effects of sunlight upon rickets and surgical tuberculosis it is excellent, and we hope it will be widely read by the laity who are just becoming dimly conscious that the doctors are realising that they are less clever than the sun. Hippocrates and other ancients no doubt recognised it long ago, but then most people don't know that !

The sequence of Dr. Saleeby's statements is not very orderly, but he makes clear what Finsen and Rollier have done, and offers well-deserved homage to Dr. Theodore Palm, who, as far back as 1890, told how systematic sun-baths would cure rickets. And now we know that a few minutes' daily exposure to sunlight will double the phosphorus in a baby's blood in a fortnight. Moreover, the terrible tale of 10,000 deaths a year in Great Britain from surgical tuberculosis ought, before long, to be numbered with "sad stories of the deaths of kings and battles long ago," now that we know that heliotherapy can do for that disease what nothing else yet has done. Nor is it to be supposed that there are not other, and perhaps wider, fields of disease in which the sun can cure. But he must have the co-operation of man if he is to do what he would—the barriers which man has set up against him must be overthrown. The smoke-screen, for instance, which hides him must be cleared away, and the results are likely to be startling. When New York forbade the production of dense smoke, in fourteen years the death-rate from consumption fell by one half. Dr. Saleeby insists that winter is the enemy because of the lack of light, and that the outstanding causes of death which have next to be conquered are "urban, hibernal, respiratory." Our climate we cannot alter, but we can alter our extraordinarily disagreeable artificial climate, though whether we should try to do so by the gas fires which Dr. Leonard Hill and Dr. Saleeby advocate is a question upon which unanimity of opinion is not to be expected. We are glad to see that Dr. Saleeby charges full tilt at that central heating which is the worst of all domestic abominations—it is "enervating and vicious." It might not be so bad if it were used in moderation, but it never is ; it is always overdone. The problem of domestic warming will not be solved until we find out how to preserve the open fire without creating volumes of smoke.

Wonderful as are the works of heliotherapy, and great as its triumphs are destined to be, it is a force not safely to be played with by the amateur or even by the medical tyro. It cannot be used by rule of thumb, and Dr. Saleeby does well to utter warnings against any attempt of that kind. In fact, he is sane and moderate throughout, and we should be glad to see his admirable handbook to this important subject on the shelves of every free library in the land.

### MIND AND HAND IN MEDICINE.

**Gastric and Duodenal Ulcer.** By Sir Berkeley Moynihan. (John Wright and Sons, Ltd. 2s. 6d. net.)

In these two lectures, delivered before the Hunterian Society, the distinguished author passes in review his experiences of the last ten years in the treatment of gastric and duodenal ulcers. As regards the diagnosis of the former condition a radiological examination has the pride of place, and next to this comes the examination of the gastric contents by the fractional method of Rehfuss. Medical treatment by rest, dieting, etc., within certain limits, may give excellent results, but in a certain percentage of cases it seems powerless to prevent relapses, whereas surgical treatment, whether by gastro-enterostomy or by gastrectomy, affords the chance of complete relief in not less than 90 per cent. of cases. The number of cases reviewed is 718, and the author has had over 500 consecutive cases treated by operation without a death.

In operative work Sir Berkeley has devoloped a routine procedure that is designed to obtain the best results—the dentist, the radiologist, and the chemist all making their "attack" upon the patient first. His power of standing an operation is next gauged, and if necessary the administration of glucose or alkali is then carried out. No haste or panic is permitted at the operation, and the most minute care is taken with regard to the after-treatment. The author concludes that "the whole treatment of these diseases is not confined to the temporary relief of symptoms, but implies also both a discovery of the near or distant cause or causes, and a removal of the structural changes which result, whenever possible." To read these lectures is to catch something of the author's enthusiasm, and few workers have shed more light upon the problems of gastric and duodenal ulcer than he.

### MAKING THE BEST OF LIFE.

**The Technique of Living.** By Harold Dearden, M.R.C.S., L.R.C.P. (Heinemann. 6s. net.)

There can never have been a time when so much advice was offered as to the details of existence. We are told how to grow thin, how to keep young, how to manage our wives, how to furnish a house, how to keep our tempers, how to make £10,000 a year, and it is open to doubt whether many of us profit very much by all this kind assistance. It is probable that most of this advice goes in at one ear and out at the other, but it cannot be denied that there are considerable numbers of people who would benefit very materially by a little sympathetic help in the ordering of their lives. They are slightly neurotic, cannot fit into their environment, find their work difficult and concentration impossible, and are always more or less tired. Now it is by people of this kind particularly that Mr. Dearden's book should be read and its contents taken to heart. He has about him something of the priest as well as the physician, and is concerned with the spirit as well as the mind and body :—

It is because so many people at the present time have been awakened to dissatisfaction with their former restricted outlook and are seeking a broader field and a bigger endeavour that so much spiritual unrest exists in every section of the community. They are finding the task too big for their untutored powers and are groping blindly in every direction for something that shall help them in their efforts. It is this perpetual struggle to adapt ourselves to certain arbitrary restrictions that is at the root of almost all forms of mental and nervous instability. Its problem is with us from the cradle to the grave, and our happiness is merely the measure of our success in solving it. Psychological treatment consists in diagnosing and solving such problems as have caused a nervous breakdown, and is of undoubted value in all cases where the patient has not become so far disorganised under the strain as to be inaccessible.

This book is not, of course, intended for the expert, who might find much to cavil at, but the ordinary man or woman who is perplexed and overburdened will find Mr. Dearden's tolerance and sanity and the essential kindliness of all he writes a great deal better than most nerve tonics.

---

### MISCELLANEOUS NOTICES.

### Learning Surgery.

Handbook of Surgery. By George L. Chiene, M.B., C.M., F.R.C.S.(Ed.), Surgeon to the Edinburgh Royal Infirmary and Lecturer on Clinical Surgery, Edinburgh University. (Edinburgh: E. and S. Livingstone. 8s.)—This small handbook should serve as an excellent introduction to surgery for those who are entering hospital for the first time. It concerns itself with the salient points in surgery without entering into the more minute details—for those students are referred to the larger text-books. Nor are detailed descriptions of operative procedures included—for those also the reader is referred to text-books on operative surgery. The book is amply illustrated, the photographs being drawn from the author's own collection. We heartily recommend it to all who are taking up the subject of surgery.

### A Handbook for the Social Worker.

Liverpool Social Workers' Handbook, 1924. (Liverpool: Gledsdale and Jennings. 2s. 6d.)—The Liverpool Council of Voluntary Aid has again issued its Social Workers' Handbook. To the people of Liverpool who are interested in the improvement of the health, character and conditions of living of their citizens, this admirably compiled booklet will show how comprehensive are the activities of its social workers, while similar workers in London would also do well to study it. As stated in the preface, "the Health Committee have established seven centres and twenty agencies throughout the city where milk is supplied to nursing mothers if required." A useful feature of the book is an index giving particulars of sources from which information can be obtained as to hospitals, dispensaries, infant care, funds in support of medical charities, relief of permanent incapacity, relief of financial distress—either temporary or permanent—insurance, industrial conditions and institutions dealing with reformation of character, social improvement and education—in fact, everything that makes for advancement among the inhabitants of a great town.

### "A Joy of Life" Novel.

Silver Star-Dust. By Cecil Adair. (Stanley Paul. 7s. 6d. net.)—Miss Cecil Adair is, we understand, the successor of Mrs. Barclay, and it is even rumoured that she is a rival of Miss Ethel Dell herself. More luscious than the former, less vigorous than the latter, she resembles them in the ability to tell a definite story, and this is apparently what the public most desires. Her stories are, it is true, not always convincing, her style is quite extraordinarily bad: educated people, for instance, are not in the habit of saying, "I'd like for so-and-so to see you." But her people are so good and kind and artless that it is not fair to be too hard on them. Still, we feel that her hero, Cosmo, needs to be repressed. At fourteen he talks in this way to a little girl, who does her share by addressing him as "Boy." "Yes, the stars are always there. And I have a little star of my own, have I not, that will always shine for me. I think it would shine on me even if I had to go down very deep into some silent land of shadow. Little star, is it so ?" And, of course, it was so.

### The Return of Cloak and Sword.

The Traveller in the Fur Cloak. By Stanley J. Weyman. (Hutchinson. 7s.)—After a long retirement from writing Mr. Stanley Weyman broke the silence some four or five years ago with "The Great House." Since then we have had "Ovington's Bank" and he now returns very completely to the style and method with which he originally won distinction. It is a welcome return. Mr. Weyman possesses in happy abundance all the essentials for a teller of romantic stories. Neither his own nor his readers' interest ever flags; he has always a surprise up his sleeve; he keeps his secrets well; he tells his tale clearly and simply; his inventive ingenuity is fecund of telling situations. He has had many imitators just as he himself received the seed from Scott and Dumas, but his followers have never bettered and rarely equalled their master. "The Traveller in the Fur Cloak" is a story of Germany at the moment of the Armistice after Wagram, and it is none the worse for being obviously founded on the mysterious disappearance of Benjamin Bathurst, the English diplomatist upon whose fate no real light has ever been shed. He vanished from sight in an instant, so to speak; so did Mr. Weyman's diplomatist, Percival. But it is the business of novelists to unravel mysteries, and after many a breathless adventure Mr. Weyman unravels his. He has produced a real cloak and sword romance in which Germany under the heel of Napoleon is reconstituted with remarkable skill and with a close knowledge of the facts. Mr. Weyman is always at his best "on the road," and here he is rarely off it.

